# Pilot Study of the Total and Phosphorylated Tau Proteins in Early-Stage Multiple Sclerosis

**DOI:** 10.3390/medicina60030416

**Published:** 2024-02-29

**Authors:** Ieva Masiulienė, Katryna Pampuščenko, Gintarė Žemgulytė, Diana Bilskienė, Vilmantė Borutaitė, Renata Balnytė

**Affiliations:** 1 Department of Neurology, Medical Academy, Lithuanian University of Health Sciences, A. Mickeviciaus Str. 9, LT-44307 Kaunas, Lithuania; gintare.zemgulyte@lsmu.lt (G.Ž.); renata.balnyte@lsmu.lt (R.B.); 2 Neuroscience Institute, Medical Academy, Lithuanian University of Health Sciences, Eiveniu Str. 4, LT-50162 Kaunas, Lithuania; katryna.pampuscenko@lsmu.lt (K.P.); vilmante.borutaite@lsmu.lt (V.B.); 3 Department of Anesthesiology, Medical Academy, Lithuanian University of Health Sciences, A. Mickeviciaus Str. 9, LT-44307 Kaunas, Lithuania; diana.bilskiene@lsmu.lt

**Keywords:** multiple sclerosis, biomarkers, neurodegeneration, cerebrospinal fluid, total tau protein, phosphorylated tau protein

## Abstract

*Background and Objectives*: Recent findings suggest that neurodegeneration starts early in the course of multiple sclerosis (MS) and significantly contributes to the progression of patients’ disability. Tau is a microtubule-binding protein that is known to play a role in the pathophysiology of many neurodegenerative disorders. Newly emerging data on tau protein-induced neurodegenerative processes and its possible involvement in MS suggest that it may be involved in the pathology of early-stage MS. Therefore, this study aimed to test this hypothesis in patients with newly diagnosed MS. *Materials and Methods*: Cerebrospinal fluid (CSF) was collected from 19 patients with newly diagnosed MS and 19 control subjects. All MS patients underwent neurological examination, lumbar punction, and brain magnetic resonance imaging (MRI). CSF concentrations of total and phosphorylated tau (phospho-tau-181) protein were measured using commercial enzyme-linked immunosorbent assay kits. *Results*: The total tau concentration was significantly higher in the CSF of MS patients compared to controls (141.67 pg/mL, IQR 77.79–189.17 and 68.77 pg/mL, IQR 31.24–109.17, *p* = 0.025). In MS patients, the total tau protein positively correlated with total CSF protein (r = 0.471, *p* = 0.048). Significantly higher total tau concentration was measured in MS patients with higher lesion load in brain MRI (≥9 versus <9 lesions; 168.33 pg/mL, IQR 111.67–222.32 and 73.33 pg/mL, IQR -32.13–139.29-, *p* = 0.021). The CSF concentration of phospho-tau-181 protein was below the detection limit in both MS and control subjects. *Conclusions*: The concentration of total tau protein level is elevated, whereas phospho-tau-181 is undetectable in the CSF of patients with early-stage MS.

## 1. Introduction

Multiple sclerosis (MS) is an autoimmune demyelinating disease of the central nervous system (CNS) with both inflammatory and neurodegenerative components [1]. It is the most common nontraumatic disabling neurological condition in young adults [1]. In recent years, the prognosis of MS patients has significantly improved owing to the development of new highly effective disease-modifying therapies (DMT). Nonetheless, expanding therapeutic options has introduced novel challenges, including the necessity for early diagnosis and vigilant monitoring of disease activity to ensure the timely initiation of DMT. In current routine clinical practice, the prognosis of MS predominantly relies on the clinical relapse rate and radiological activity of the disease [2]. However, these characteristics predominantly represent an ongoing neuroinflammation. Meanwhile, neurodegeneration has been considered the driving force of disability progression [3,4].

Among various CNS proteins, the tau protein has been established to play an important role in the pathophysiology of neurodegenerative disorders [5,6]. Tau is a soluble protein encoded by the microtubule-associated protein tau (*MAPT*) gene [7]. It is primarily found in the axons of neurons and at lower levels in glial cells, in particular oligodendrocytes and astrocytes [7]. The main role of the tau protein is the stabilization of microtubule bundles; however, it also plays a role in axonal transport, synaptic transmission, synaptic plasticity, and adult neurogenesis [5,8,9]. The normal function of the tau protein may be impaired by various mutations in the *MAPT* gene and post-translational modifications, including phosphorylation and truncation [9,10]. Regarding phosphorylation, tau protein has multiple phosphorylation sites; however, only some of them, including phospho-tau-181, are currently considered to have prognostic value in neurodegenerative processes [11]. These pathological modifications result in tau aggregation in the neuronal somata leading to the development of neurodegenerative disorders collectively called tauopathies such as Alzheimer’s disease, corticobasal degeneration, progressive supranuclear palsy, frontotemporal dementia, and others [5,6,9].

Previous studies of tauopathies showed an increased concentration of total tau protein and its phosphorylated form in the cerebrospinal fluid (CSF) of these patients [12,13,14]. At first, these findings were considered to be associated mainly with the passive release of tau protein from degenerating and dying neurons [15]. However, recent research showed that tau can be secreted from neurons independently of cell death [7,15,16]. Moreover, tau protein can spread from one neuron to another inducing misfolding and aggregation of intracellular tau protein in previously healthy neurons [15]. This intercellular transfer can be mediated by several different mechanisms including exosomes, ectosomes, tunnelling nanotubes acting as intercellular bridges, heparan sulphate proteoglycan-mediated macropinocytosis of tau seeds, and binding to muscarinic or synaptic receptors [15,17,18]. Experimental research in animal models also showed that the injection of patient brain-derived tau aggregates into the mouse brain induces pathological changes that match both the structural features and cell-type specificity of the tau pathology of the donor tauopathy [10]. Although this pathological spreading of tau protein, also called seeding, was investigated in detail in tauopathies, mostly Alzheimer’s disease, recently it was also demonstrated in MS patients. LaCroix et al. [19] showed tau seeding in the brain homogenates from six out of eight MS cases, suggesting tau may act as a mediator of neurodegeneration in MS.

Recent research has also provided evidence that pathological forms of tau protein may also impair myelination, affecting oligodendrocytes. In these cells, tau protein is expressed in its fully matured form, after the peak of myelin development [20]. Expressing a truncated form of tau protein in oligodendrocytes can elicit myelin decrease and gait abnormalities in mice models [21]. Moreover, the pathological forms of tau protein may be imported to oligodendrocytes from the neurons they envelop, leading to apoptosis [22]. In aging mice, the spread of phospho-tau from neurons to oligodendrocytes leads to impairment of action potential propagation [23]. In a mouse model of acute demyelination mimicking MS, tau-positive oligodendrocytes were absent in the corpus callosum [20]. These findings suggest that tau protein can also be involved in the disruption of oligodendrocytes, which are an essential part of MS pathology.

In general, MS is considered to be a two-stage disease that begins with demyelination leading to neuronal degeneration; however, recent findings suggest that early neurodegenerative processes are already present at the initial disease stages [24]. Various imaging biomarkers of early degeneration were previously established using advanced technologies such as brain and spinal cord atrophy on magnetic resonance imaging (MRI) and thinning of the retinal nerve fibre and ganglion cell layers on optical coherence tomography [25,26,27]. However, these studies are currently of limited availability in routine clinical practice. As the majority of MS patients undergo lumbar puncture (LP) during the initial investigation, the CSF may be a convenient diagnostic material directly reflecting early pathological processes occurring in the CNS. Newly emerging data on tau protein’s role in neurodegeneration and disruption of both neurons and raises the question of whether tau protein-related pathology might be present in the early stages of the disease and contribute to early neurodegeneration in MS.

To investigate this hypothesis, we examined concentrations of total and phospho-tau-181 protein levels in the CSF of patients newly diagnosed with MS. We then analysed the potential correlations between these biomarkers and various demographic, clinical, and radiological characteristics of our patient cohort.

## 2. Materials and Methods

### 2.1. Patients and CSF Collection

At the Lithuanian University of Health Sciences Hospital Kauno Klinikos, Department of Neurology, we enrolled 19 newly diagnosed MS patients. They all underwent clinical assessment, brain MRI, and LP. The diagnosis of MS was made according to the 2017 revised McDonald criteria [28]. At the time of investigation, 12 of them had a relapse defined as an episode of neurological disturbance that lasted for at least 24 h and could not be better explained by another cause [28]. None of the patients were treated with glucocorticoids or DMT before CSF sampling. After LP, intravenous methylprednisolone therapy was administrated for 12 patients experiencing a relapse. Disability was scored with the Expanded Disability Status Scale (EDSS) at the time of LP and after six months. At the six-month follow-up, twelve patients were diagnosed with relapsing-remitting MS, while seven were still under observation of the clinical course of MS. Symptom duration was calculated from the date of the first MS symptom that the patient recalled. Brain MRI was performed for all patients. A spinal (either cervical or thoracic) MRI was performed in the case of clinical suspicion of spinal cord lesions. An MRI was performed not earlier than 4 months before LP (median time 24 days). We recorded T2 white matter lesion load (LL) with a cut-off of nine lesions to define high and low LL [29], their localization, the presence of spinal lesions, gadolinium-enhancing (Gd+) lesions, and atrophy (visible on conventional MRI as widening of convexity subarachnoid spaces in two patients and shrinkage of spinal cord anterior and medial white matter columns in one patient).

Control CSF samples were obtained from 19 patients. Thirteen of them underwent LP due to the investigation of various noninflammatory neurological disorders (NINDs). Their final clinical diagnoses included tension-type headache (*n* = 6), abducens nerve palsy (*n* = 1), cerebellar ataxia (*n* = 1), polyneuropathy (*n* = 1), narcolepsy (*n* = 1), residual non-specific CNS lesions after neuroinfection in childhood (*n* = 1), and subjective neurological symptoms (*n* = 2). For the last six control subjects, CSF collection occurred before the administration of spinal anaesthesia. This procedure was conducted as a preparation for microdiscectomy at the L4/5 or L5/S1 spinal levels.

This study was approved by the Kaunas Regional Biomedical Research Ethics Committee (No. BE-2-61). At the time of LP, all patients signed informed consent forms, thereby providing their agreement for both the intervention and CSF collection for research purposes.

### 2.2. CSF Analysis and Biomarkers Identification

The LP was performed in the L3/4 or L4/5 interspaces and CSF was collected for routine analysis and biobanking. The routine laboratory tests for MS patients included total and differential white blood cell (WBC) count, total protein, albumin, immunoglobulin G (IgG), and glucose concentrations as well as oligoclonal bands (OGB). IgG index was also evaluated. For biobanking, the CSF was collected in polypropylene tubes and then centrifuged at 2000× *g* for 10 min at room temperature. After spinning, samples were divided into aliquots and frozen for storage at −80 °C until analysis.

Concentrations of total and phospho-tau-181 in CSF were measured using commercially available enzyme-linked immunosorbent assay (ELISA) kits: Human Total Tau (cat #KHB0041) and Human Tau [pT181] phosphoELISA™ (cat #KHO0631) from Invitrogen, ThermoFisher Scientific (Waltham, Massachusetts, USA). For one measurement, 50 μL and 25 μL of CSF were used for total tau and phosho-tau analysis, respectively. Measurements were taken in duplicate according to the manufacturer’s protocols. Absorbance was measured with a Multiskan GO plate reader (Thermo Fisher Scientific, Waltham, MA, USA).

### 2.3. Statistics

Statistical analyses were performed using SPSS 26.0 (SPSS Inc., Chicago, IL, USA). Data visualization was performed using RStudio (version 2023.12.0+369, R Core Team, 2020) with ggplot2 package (v3.4.4; [30]). The normality distribution of data was tested with the Shapiro–Wilk test. Variables with normal distribution were presented as mean ± standard error (SEM), and statistical comparisons between two groups were performed using independent samples *T*-Test. Non-normally distributed variables were presented as the median and interquartile range (IQR) [Q1–Q3]. Statistical comparisons between two groups were performed using the Mann–Whitney U test, whereas the Kruskal–Wallis test was applied for the comparison of three or more groups. The distribution by gender between MS and control patients was tested using the Chi-Square test. Correlation analysis was performed using the Spearman correlation test. The *p*-value was considered statistically significant when *p* < 0.05.

## 3. Results

The control patients did not differ from MS patients according to sex (*p* = 0.732) and age (35.42 ± 2.59 and 36.11 ± 2.60, respectively, *p* = 0.853). Among MS patients, the most common clinical expressions of MS were pyramidal (47.36%) and sensory (42.10%) symptoms. The median EDSS at the diagnosis was 2.5 (range 1.0–5.5). The majority of patients exhibited nine or more lesions on their brain MRI scans (68.42%). Additionally, five patients (26.32%) displayed active gadolinium-enhancing brain lesions. The detailed characteristics of the MS patients are provided in Table 1.

In the routine CSF analysis of MS patients, only low-level CSF cytosis was identified with a predominance of monomorphonuclear WBC (4, IQR (1–8), and 3, IQR (1–7), respectively). The majority of patients had elevated IgG index (0.81, IQR –0.70–0.94-). The majority of MS patients were positive for oligoclonal bands in the CSF but not in the serum (type 2 pattern; *n* = 17), while two patients had oligoclonal bands in both serum and CSF with additional bands in CSF (type 3 pattern). The detailed characteristics of the CSF analytes of the MS patients are presented in Table 2.

**Figure 1:**
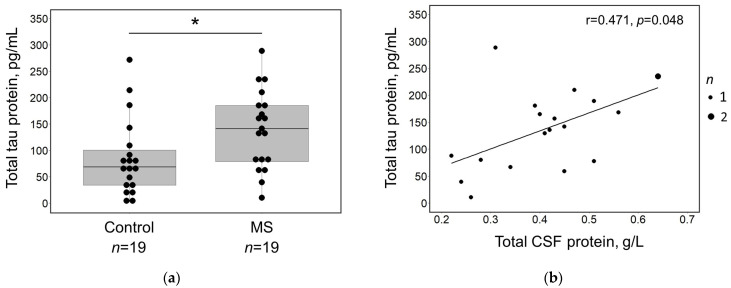
(**a**) Box plot illustrating the distribution of total tau concentrations in the CSF of the MS patients (*n* = 19) and controls (*n* = 19). A statistically significant difference between groups is marked with an asterisk (*). Black dots indicate individual data points. (**b**) Scatterplot of total tau protein concentrations in the CSF plotted against the corresponding total protein concentration in the CSF. The larger dot indicates two overlapping data points. (**a**,**b**) Measurements were taken in duplicate according to the manufacturer’s protocols. Abbreviations: MS—multiple sclerosis; CSF—cerebrospinal fluid.

The total tau protein was detected in all analysed CSF samples. Its concentration did not differ between genders or correlate with the patient’s age. Significantly higher total tau concentration was measured in MS patients compared to controls (141.67 pg/mL, IQR 77.79–189.17, and 68.77 pg/mL, IQR 31.24–109.17, *p* = 0.025; Figure 1a).

In MS patients, there was a positive correlation between the concentrations of total tau protein and total CSF protein (r = 0.471, *p* = 0.048; Figure 1b). No relation between the total tau protein and other routine CSF tests (differential WBC count, albumin, IgG, and IgG index) was observed.

Among different clinical MS characteristics, a lower total tau protein concentration was found in patients with sensory symptoms compared with the intact sensory system (83.75 pg/mL, IQR 60.80–149.79, and 180.83 pg/mL, IQR 135.83–234.63-, *p* = 0.033).

**Figure 2:**
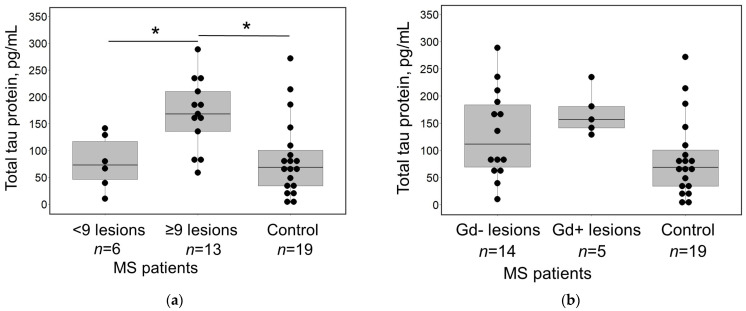
(**a**) Box plot illustrating the distribution of CSF tau between MS patients with <9 (*n* = 6) and ≥9 lesions (*n* = 13) in the brain MRI as well as control subjects. A statistically significant difference between groups is marked with an asterisk (*). (**b**) Box plot illustrating the distribution of CSF tau between MS patients with and without Gd+ lesions (*n* = 5 and *n* = 14, respectively) as well as control subjects (*n* = 19). Black dots indicate individual data points. (**a**,**b**) Measurements were taken in duplicate according to the manufacturer’s protocols. Abbreviations: MS—multiple sclerosis; Gd—gadolinium.

Significantly higher total tau concentration was measured in MS patients with higher LL in brain MRI (≥9 versus <9 lesions; 168.33 pg/mL, IQR 111.67–222.32, and 73.33 pg/mL, IQR 32.13–139.29, *p* = 0.021). Moreover, in comparison with control patients, total tau concentration significantly differed only from MS patients with ≥9 lesions (68.77 pg/mL, IQR 31.24–109.17, and 168.33 pg/mL, IQR 111.67–222.32, *p* = 0.003; Figure 2a). Given the correlation between total tau concentration and total CSF protein, we computed the normalized total tau concentration (i.e., total tau concentration divided by total CSF protein concentration). Repeated statistical analysis showed that a difference between total tau concentration and brain LL remained significant after correction for total CSF protein (≥9 versus <9 lesions; 367.19, IQR 312.00–429.65, and 240.90, IQR 133.05–314.87, *p* = 0.017).

Other clinical features (type of clinical symptoms, their duration, and presence of relapse), MRI characteristics (lesions localisation, presence of Gd+ lesions, and brain atrophy), and EDSS scores did not correlate with total tau levels.

The CSF concentration of phospho-tau-181 protein was below the detection limit in both MS patients and control subjects.

## 4. Discussion

In this study, we investigated the role of tau protein in the early stage of MS. We aimed to determine both total and phospho-tau-181 protein concentrations in the CSF collected during the initial investigation for MS diagnostics. Furthermore, we attempted to establish a relationship between the determined total tau concentration and various clinical and radiological patient characteristics. Our findings revealed an increased concentration of total tau in MS patients as compared to the control subjects. Moreover, the total tau concentration was significantly elevated in the subset of patients exhibiting a higher LL (≥9 lesions) in brain MRI and this finding remained significant after the correction of total tau concentration in regard to total CSF protein. However, phospho-tau-181 was undetectable in all patients.

Total tau protein, as an MS biomarker, has been previously investigated; however, studies have yielded contradictory data. Our findings are consistent with the research demonstrating an elevated concentration of total tau in patients with MS [31,32,33,34,35,36,37], whereas other studies showed no difference in tau concentrations between patients with MS and control subjects [38,39,40,41,42,43,44]. However, the recent meta-analysis summarizing the majority of these studies concluded that MS patients show higher total tau protein levels compared to controls [45]. Despite that, data remain controversial comparing total tau protein levels in different stages of MS. Colucci et al. [46] revealed a statistically significant difference only between clinically isolated syndrome patients and controls. Virgilio et al. [47] showed that CSF tau level may be a predictor of early disability in MS. Interestingly, two studies showed a significant decrease in total tau in CSF of the patients with secondary progressive MS [34,48]. Their authors hypothesized that tau concentration in the CSF of patients with MS decreases over the course of the disease, potentially reflecting the degree of parenchymal brain loss [48]. Therefore, a diminished neuronal population in the later stages of MS results in a depletion of tau resources and lower levels of tau in the CSF [34]. In our study, patients were included at the time of diagnosis and the median duration of the symptoms calculated from the first complaints the patients recalled was 6 months. Therefore, an observed elevation in tau protein levels within our patient population may be indicative of an ongoing neuronal injury within previously intact brain tissue.

In our study, we did not find significant relations between CSF tau protein concentration and signs of inflammatory disease activity such as clinical relapse and the presence of Gd+ lesions in brain MRI. It is consistent with meta-analysis data showing that total tau did not differ between MS patients in relapse and remission [45]. However, data regarding the association between tau protein and the radiological activity of MS are less abundant and quite controversial [35,36,43,47,49]. Regarding routine CSF analytes, our findings indicate a positive correlation between total tau and total CSF protein concentrations. Although an elevated total protein concentration can represent blood–brain barrier (BBB) disruption, it is not specific for inflammatory activity and is within a normal range in the majority of MS patients [50]. Furthermore, our study revealed that total tau did not exhibit significant correlations with either CSF leukocyte count or the IgG index, the latter being a more specific indicator for MS as it reflects not only BBB dysfunction but also intrathecal production of immunoglobulins [51]. In summary, these findings suggest that the elevation in total tau levels observed in MS patients is unlikely to be attributed to acute inflammation.

In our study, CSF tau protein concentration was significantly elevated in patients with a higher T2 LL in the brain MRI. Moreover, additional analysis of normalized tau protein concentration in these patients revealed that this finding is independent of total CSF protein. Our findings align with several previous studies that also demonstrated a positive association between CSF total tau concentration and T1 and T2 LL [36,39]. However, other authors did not report such a relationship in their studies [43,47,49]. Despite discrepancies in the literature, a higher LL may also represent a higher perilesional area that is characterized by ongoing neurodegenerative injury as shown by both histological and MRI studies [52,53].

Phosphorylation of tau protein is the most studied post-translational modification of this protein related to neurodegenerative processes [15]. To test whether phospho-tau is involved in the pathogenesis of early MS, we examined its concentration in the CSF of our patients. However, phospho-tau-181 was not detected in the samples of either MS patients or control subjects. According to post-mortem studies, tau hyperphosphorylation is found in the brain samples from patients with early aggressive, primary progressive, and secondary progressive MS, whereas the insoluble form is present only in cases of progressive MS [54,55,56]. It implies that the accumulation of phospho-tau protein may be the cause of progressive MS and it may be a secondary tauopathy [19]. In our study, the CSF was collected at the beginning of the disease and the majority of patients showed minimal disability. Therefore, it is most likely that phosphorylation of tau protein plays a role in MS pathophysiology in the later stages of the disease as well as the most aggressive forms of early MS. However, a few other studies have detected phospho-tau in the CSF of MS patients; nevertheless, their data are conflicting. While some have suggested a tendentious elevation of phospho-tau in RR MS patients during relapse [44], others have shown the correlation of phospho-tau-181 with longer disease duration but not disease activity [32]. Therefore, longitudinal studies are required to reveal at which stage of the disease tau phosphorylation may occur.

It is important to note that, in the context of neurodegeneration, extracellular tau can directly activate microglia, causing neuronal damage through enhanced phagocytosis [57]. Disruptions in synaptic phagocytosis can contribute to neurodegenerative diseases and cognitive decline through excessive loss of functional synapses. Furthermore, microglia can recognize and engulf stressed-but-viable neurons, leading to their death by primary phagocytosis, also known as phagoptosis. Increasing evidence suggests that aberrant phagocytosis of live synapses and neurons contributes to pathological processes in animal models of Alzheimer’s disease, Parkinson’s disease, primary tauopathies, and retinal degeneration, as well as after ischemia and during aging [57,58]. Our previous research has demonstrated that extracellular tau protein can cause microglia-dependent phosphatidylserine exposure on the external plasma membrane leaflet of living neurons, which serves as an “eat-me” signal for phagocytes, leading to phagocytosis [59]. Moreover, we found that extracellular tau can activate microglia via Toll-like 4 receptors and promote neuronal phagocytosis through phagocytic phosphatidylserine recognizing MerTK receptors and purinergic P2Y6 receptors [59,60,61]. Other studies have shown that microglial engulfment of complement-labelled synapses leads to synaptic pathology in a murine tauopathy model [62]. Notably, in MS, synapse dysfunctions occur early and are independent of demyelination [63,64]. Moreover, post-mortem studies show that neuronal loss in the MS brain could also occur independently of demyelination [65]. In addition, it was found that in MS patients’ hippocampi and visual thalamus, microglia can engulf and eliminate complement-labelled synapses [66,67]. Experiments with MS-relevant animal models also confirm that phagocyte-mediated synapse engulfment and loss occur early in the disease [66,67]. Thus, we can speculate that in MS, elevated levels of circulating tau at early disease stages could contribute to synaptic damage and microglial activation in MS, leading to neurodegenerative processes. However, this hypothesis requires further investigation.

This study has several limitations. The data presented in this article are part of a pilot study investigating CSF proteins that may be related to neurodegeneration in patients with early-stage MS at the time of diagnosis. Before collecting a larger cohort of patients and conducting a more comprehensive analysis of selected biomarkers, our objective was to identify promising proteins for further investigation. Therefore, the included sample size was small and the follow-up period was only six months. Because of the latter reason, the clinical course of the disease has not yet been determined in approximately one-third of MS patients. Moreover, our control groups were heterogeneous, including various NINDs and patients with compressive radiculopathy undergoing microdiscectomy. We cannot decline that tau protein may also play a role in some patients with NINDs as well.

## 5. Conclusions

In conclusion, our findings showed that total tau protein level is elevated, while phospho-tau-181 is undetectable in the CSF of patients with early-stage MS. An analysis of the total tau concentration among MS patients, stratified by their clinical and radiological characteristics, unveiled a significant elevation in CSF total tau protein levels in individuals exhibiting a higher T2 LL on brain MRI. Hence, our findings suggest that the total tau concentration may serve as an indicator of the cumulative neuronal injury observed in patients with MS. However, these results need to be replicated in a larger sample of patients with early-stage MS. Furthermore, further studies elucidating the molecular mechanisms underlying tau protein-related pathology in MS are imperative for a comprehensive understanding of its pathological significance in this disorder.

## Figures and Tables

**Table 1:** Demographic, clinical, and MRI characteristics of MS patients. Abbreviations: EDSS—Expanded Disability Status Scale; MRI—magnetic resonance imaging; MS—multiple sclerosis; Gd—gadolinium. Data are presented as median (IQR or range) or percentage (total number).

Demographic Characteristics	MS Patients (*n* = 19)
Age, median (IQR)	32 (8–44)
Gender (female), *n* (%)	12 (63.16%)
Symptoms duration (months), median (IQR)	6 (2–18)
**Clinical characteristics**	
Clinical symptoms	
Pyramidal, % (*n*)	47.36% (9)
Sensory, % (*n*)	42.10% (8)
Brainstem/cerebellum, % (*n*)	31.58% (6)
Optic neuritis, % (*n*)	26.32% (5)
Myelitis, % (*n*)	5.26% (1)
EDSS	
At diagnosis, median (range)	2.5 (1.0–5.5)
At six-month follow-up, median (range)	1.5 (1.0–6.0)
The clinical course of MS	
Relapsing-remitting MS, % (*n*)	63.16% (12)
Not yet determined, % (*n*)	36.84% (7)
**MRI characteristics**	
Lesions load in the brain MRI	
<9 lesions, % (*n*)	31.58% (6)
≥9 lesions, % (*n*)	68.42% (13)
Lesions localization	
Periventricular, % (*n*)	100% (19)
Juxta-, subcortical, % (*n*)	84.21% (16)
Corpus callosum, % (*n*)	89.47% (17)
Brainstem and cerebellum, % (*n*)	94.74% (18)
Optic nerve, % (*n*)	21.05% (4)
Spinal cord, % (*n*)	68.42% (13)
Gd+ lesions, % (*n*)	26.32% (5)
Atrophy, % (*n*)	15.79% (3)

**Table 2:** Routine CSF analytes in MS patients. Abbreviations: CSF—cerebrospinal fluid; IgG—immunoglobulin G; MS—multiple sclerosis; WBC—white blood cells. Data are presented as median (IQR) or percentage (total number).

Routine CSF Analytes	MS Patients (*n* = 19)	Reference Range
Total WBC count, ×106/L	4 (1–8)	0–5
Polymorphonuclear WBC, ×106/L	0 (0–0)	–
Monomorphonuclear WBC, ×106/L	3 (1–7)	–
Total CSF protein, g/L	0.42 (0.30–0.48)	0.15–0.45
Glucose, mmol/L	3.31 (3.13–3.70)	2.2–3.9
Albumin, mg/L	234 (183–263)	139–246
Immunoglobulin G, mg/L	48.2 (32.7–66.6)	4.8–58.6
IgG index	0.81 (0.70–0.94)	–
Oligoclonal bands, % (*n*)	100% (19)	–

## Data Availability

The datasets generated during the current study are available from the corresponding author upon reasonable request.

## References

[1] Ward M., Goldman M.D. (2022). Epidemiology and Pathophysiology of Multiple Sclerosis. Contin. Lifelong Learn. Neurol..

[2] Zhang Y., Cofield S., Cutter G., Krieger S., Wolinsky J.S., Lublin F. (2022). Predictors of Disease Activity and Worsening in Relapsing-Remitting Multiple Sclerosis. Neurol. Clin. Pract..

[3] Eshaghi A., Kievit R.A., Prados F., Sudre C.H., Nicholas J., Cardoso M.J., Chan D., Nicholas R., Ourselin S., Greenwood J. (2019). Applying causal models to explore the mechanism of action of simvastatin in progressive multiple sclerosis. Proc. Natl. Acad. Sci. USA.

[4] Harari G., Gurevich M., Dolev M., Falb R.Z., Achiron A. (2023). Faster progression to multiple sclerosis disability is linked to neuronal pathways associated with neurodegeneration: An ethnicity study. PLoS ONE.

[5] Guo T., Noble W., Hanger D.P. (2017). Roles of tau protein in health and disease. Acta Neuropathol..

[6] Liang S.Y., Wang Z.T., Tan L., Yu J.T. (2022). Tau Toxicity in Neurodegeneration. Mol. Neurobiol..

[7] Didonna A. (2020). Tau at the interface between neurodegeneration and neuroinflammation. Genes Immun..

[8] Barbier P., Zejneli O., Martinho M., Lasorsa A., Belle V., Smet-Nocca C., Tsvetkov P.O., Devred F., Landrieu I. (2019). Role of tau as a microtubule-associated protein: Structural and functional aspects. Front. Aging Neurosci..

[9] Caamaño-Moreno M., Gargini R. (2023). Tauopathies: The Role of Tau in Cellular Crosstalk and Synaptic Dysfunctions. Neuroscience.

[10] Robert A., Schöll M., Vogels T. (2021). Tau seeding mouse models with patient brain-derived aggregates. Int. J. Mol. Sci..

[11] Trushina N.I., Bakota L., Mulkidjanian A.Y., Brandt R. (2019). The Evolution of Tau Phosphorylation and Interactions. Front. Aging Neurosci..

[12] Olsson B., Lautner R., Andreasson U., Öhrfelt A., Portelius E., Bjerke M., Hölttä M., Rosén C., Olsson C., Strobel G. (2016). CSF and blood biomarkers for the diagnosis of Alzheimer’s disease: A systematic review and meta-analysis. Lancet Neurol..

[13] Schoonenboom N., Reesink F., Verwey N., Kester M., Teunissen C., van de Ven P., Pijnenburg Y.A., Blankenstein M.A., Rozemuller A.J., Scheltens P. (2012). Cerebrospinal fluid markers for differential dementia diagnosis in a large memory clinic cohort. Neurology.

[14] Urakami K., Wada K., Arai H., Sasaki H., Kanai M., Shoji M., Ishizu H., Kashihara K., Yamamoto M., Tsuchiya-Ikemoto K. (2001). Diagnostic significance of tau protein in cerebrospinal fluid from patients with corticobasal degeneration or progressive supranuclear palsy. J. Neurol. Sci..

[15] Brunello C.A., Merezhko M., Uronen R.L., Huttunen H.J. (2020). Mechanisms of secretion and spreading of pathological tau protein. Cell Mol. Life Sci..

[16] Kanmert D., Cantlon A., Muratore C.R., Jin M., O’Malley T.T., Lee G., Young-Pearse T.L., Selkoe D.J., Walsh D.M. (2015). C-terminally truncated forms of tau, but not full-length tau or its C-terminal fragments, are released from neurons independently of cell death. J. Neurosci..

[17] Zhang H., Cao Y., Ma L., Wei Y., Li H. (2021). Possible Mechanisms of Tau Spread and Toxicity in Alzheimer’s Disease. Front. Cell Dev. Biol..

[18] Han Z.Z., Kang S.G., Arce L., Westaway D. (2023). Prion-like strain effects in tauopathies. Cell Tissue Res..

[19] LaCroix M.S., Mirbaha H., Shang P., Zandee S., Foong C., Prat A., White C.L., Stuve O., Diamond M.I. (2022). Tau seeding in cases of multiple sclerosis. Acta Neuropathol. Commun..

[20] Torii T., Miyamoto Y., Nakata R., Higashi Y., Shinmyo Y., Kawasaki H., Miyasaka T., Misonou H. (2023). Identification of Tau protein as a novel marker for maturation and pathological changes of oligodendrocytes. Glia.

[21] LoPresti P. (2015). Inducible Expression of a Truncated Form of Tau in Oligodendrocytes Elicits Gait Abnormalities and a Decrease in Myelin: Implications for Selective CNS Degenerative Diseases. Neurochem. Res..

[22] Torii T. (2024). Abnormal expression of Tau in damaged oligodendrocytes of HLD1 mice. Neural Regen. Res..

[23] Viney T.J., Sarkany B., Ozdemir A.T., Hartwich K., Schweimer J., Bannerman D., Somogyi P. (2022). Spread of pathological human Tau from neurons to oligodendrocytes and loss of high-firing pyramidal neurons in aging mice. Cell Rep..

[24] Mey G.M., Mahajan K.R., DeSilva T.M. (2023). Neurodegeneration in multiple sclerosis. WIREs Mech. Dis..

[25] Kuhlmann T., Moccia M., Coetzee T., Cohen J.A., Correale J., Graves J., Marrie R.A., Montalban X., Yong V.W., Thompson A.J. (2023). Multiple sclerosis progression: Time for a new mechanism-driven framework. Lancet Neurol..

[26] Paul F., Calabresi P.A., Barkhof F., Green A.J., Kardon R., Sastre-Garriga J., Schippling S., Vermersch P., Saidha S., Gerendas B.S. (2021). Optical coherence tomography in multiple sclerosis: A 3-year prospective multicenter study. Ann. Clin. Transl. Neurol..

[27] Mey G.M., DeSilva T.M. (2023). Utility of the visual system to monitor neurodegeneration in multiple sclerosis. Front. Mol. Neurosci..

[28] Thompson A.J., Banwell B.L., Barkhof F., Carroll W.M., Coetzee T., Comi G., Correale J., Fazekas F., Filippi M., Freedman M.S. (2018). Diagnosis of multiple sclerosis: 2017 revisions of the McDonald criteria. Lancet Neurol..

[29] Minneboo A., Barkhof F., Polman C.H., Uitdehaag B.M.J., Knol D.L., Castelijns J.A. (2004). Infratentorial Lesions Predict Long-term Disability in Patients with Initial Findings Suggestive of Multiple Sclerosis. Arch. Neurol..

[30] Wickham H. (2016). Ggplot2: Elegant graphics for data analysis.

[31] Kapaki E., Paraskevas G.P., Michalopoulou M., Kilidireas K. (2000). Increased Cerebrospinal Fluid Tau Protein in Multiple Sclerosis. Eur. Neurol..

[32] Bartosik-Psujek H., Stelmasiak Z. (2006). The CSF levels of total-tau and phosphotau in patients with relapsing-remitting multiple sclerosis. J. Neural Transm..

[33] Bartosik-Psujek H., Psujek M., Jaworski J., Stelmasiak Z. (2011). Total tau and S100b proteins in different types of multiple sclerosis and during immunosuppressive treatment with mitoxantrone. Acta Neurol. Scand..

[34] Kosehasanogullari G., Ozakbas S., Idiman E. (2015). Tau protein levels in the cerebrospinal fluid of the patients with multiple sclerosis in an attack period: Low levels of tau protein may have significance, too. Clin. Neurol. Neurosurg..

[35] Brettschneider J., Maier M., Arda S., Claus A., Süssmuth S.D., Kassubek J., Tumani H. (2005). Tau protein level in cerebrospinal fluid is increased in patients with early multiple sclerosis. Mult. Scler..

[36] Brettschneider J., Petzold A., Junker A., Tumani H. (2006). Axonal damage markers in the cerebrospinal fluid of patients with clinically isolated syndrome improve predicting conversion to definite multiple sclerosis. Mult. Scler..

[37] Terzi M., Birinci A., Çetinkaya E., Onar M.K. (2007). Cerebrospinal fluid total tau protein levels in patients with multiple sclerosis. Acta Neurol. Scand..

[38] Sladkova V., Mareš J., Lubenova B., Zapletalova J., Stejskal D., Hlustik P., Kanovsky P. (2011). Degenerative and inflammatory markers in the cerebrospinal fluid of multiple sclerosis patients with relapsing-remitting course of disease and after clinical isolated syndrome. Neurol. Res..

[39] Pietroboni A.M., Schiano Di Cola F., Scarioni M., Fenoglio C., Spanò B., Arighi A., Cioffi S.M., Oldoni E., De Riz M.A., Basilico P. (2017). CSF β-amyloid as a putative biomarker of disease progression in multiple sclerosis. Mult. Scler..

[40] Jiménez-Jiḿnez F.J., Zurdo J.M., Hernánz A., Medina-Acebrón S., de Bustos F., Barcenilla B., Sayed Y., Ayuso-Peralta L. (2002). Tau protein concentrations in cerebrospinal fluid of patients with multiple sclerosis. Acta Neurol. Scand..

[41] Guimarães J., Cardoso M.J., Sá M.J. (2006). Tau protein seems not to be a useful routine clinical marker of axonal damage in multiple sclerosis. Mult. Scler..

[42] Teunissen C.E., Iacobaeus E., Khademi M., Brundin L., Norgren N., Koel-Simmelink M.J.A., Schepens M., Bouwman F., Twaalfhoven H.A., Blom H.J. (2009). Combination of CSF N-acetylaspartate and neurofilaments in multiple sclerosis. Neurology.

[43] Mori F., Rossi S., Sancesario G., Codecá C., Mataluni G., Monteleone F., Buttari F., Kusayanagi H., Castelli M., Motta C. (2011). Cognitive and cortical plasticity deficits correlate with altered amyloid-Β CSF levels in multiple sclerosis. Neuropsychopharmacology.

[44] Szalardy L., Zadori D., Simu M., Bencsik K., Vecsei L., Klivenyi P. (2013). Evaluating biomarkers of neuronal degeneration and neuroinflammation in CSF of patients with multiple sclerosis—Osteopontin as a potential marker of clinical severity. J. Neurol. Sci..

[45] Momtazmanesh S., Shobeiri P., Saghazadeh A., Teunissen C.E., Burman J., Szalardy L., Klivenyi P., Bartos A., Fernandes A., Rezaei N. (2021). Neuronal and glial CSF biomarkers in multiple sclerosis: A systematic review and meta-analysis. Rev. Neurosci..

[46] Colucci M., Roccatagliata L., Capello E., Narciso E., Latronico N., Tabaton M., Mancardi G.L. (2004). The 14-3-3 protein in multiple sclerosis: A marker of disease severity. Mult. Scler..

[47] Virgilio E., Vecchio D., Crespi I., Serino R., Cantello R., Dianzani U., Comi C. (2021). Cerebrospinal Tau levels as a predictor of early disability in multiple sclerosis. Mult. Scler. Relat. Disord..

[48] Jaworski J., Psujek M., Janczarek M., Szczerbo-Trojanowska M., Bartosik-Psujek H. (2012). Total-tau in cerebrospinal fluid of patients with multiple sclerosis decreases in secondary progressive stage of disease and reflects degree of brain atrophy. Ups. J. Med. Sci..

[49] Virgilio E., Vecchio D., Crespi I., Puricelli C., Barbero P., Galli G., Cantello R., Dianzani U., Comi C. (2022). Cerebrospinal fluid biomarkers and cognitive functions at multiple sclerosis diagnosis. J. Neurol..

[50] Deisenhammer F., Zetterberg H., Fitzner B., Zettl U.K. (2019). The cerebrospinal fluid in multiple sclerosis. Front. Immunol..

[51] Ziemssen T., Akgün K., Brück W. (2019). Molecular biomarkers in multiple sclerosis. J. Neuroinflammation.

[52] Oost W., Huitema A.J., Kats K., Giepmans B.N.G., Kooistra S.M., Eggen B.J.L., Baron W. (2023). Pathological ultrastructural alterations of myelinated axons in normal appearing white matter in progressive multiple sclerosis. Acta Neuropathol. Commun..

[53] Granberg T., Fan Q., Treaba C.A., Ouellette R., Herranz E., Mangeat G., Louapre C., Cohen-Adad J., Klawiter E.C., Sloane J.A. (2017). In vivo characterization of cortical and white matter neuroaxonal pathology in early multiple sclerosis. Brain.

[54] Anderson J.M., Hampton D.W., Patani R., Pryce G., Crowther R.A., Reynolds R., Franklin R.J., Giovannoni G., Compston D.A., Baker D. (2008). Abnormally phosphorylated tau is associated with neuronal and axonal loss in experimental autoimmune encephalomyelitis and multiple sclerosis. Brain.

[55] Anderson J.M., Patani R., Reynolds R., Nicholas R., Compston A., Spillantini M.G., Chandran S. (2009). Evidence for abnormal tau phosphorylation in early aggressive multiple sclerosis. Acta Neuropathol..

[56] Anderson J.M., Patani R., Reynolds R., Nicholas R., Compston A., Spillantini M.G., Chandran S. (2010). Abnormal tau phosphorylation in primary progressive multiple sclerosis. Acta Neuropathol..

[57] Brown G.C. (2023). Cell death by phagocytosis. Nat. Rev. Immunol..

[58] Butler C.A., Popescu A.S., Kitchener E.J.A., Allendorf D.H., Puigdellívol M., Brown G.C. (2021). Microglial phagocytosis of neurons in neurodegeneration, and its regulation. J. Neurochem..

[59] Pampuscenko K., Morkuniene R., Sneideris T., Smirnovas V., Budvytyte R., Valincius G., Brown G.C., Borutaite V. (2020). Extracellular tau induces microglial phagocytosis of living neurons in cell cultures. J. Neurochem..

[60] Pampuscenko K., Morkuniene R., Krasauskas L., Smirnovas V., Brown G.C., Borutaite V. (2023). Extracellular tau stimulates phagocytosis of living neurons by activated microglia via Toll-like 4 receptor–NLRP3 inflammasome–caspase-1 signalling axis. Sci. Rep..

[61] Puigdellívol M., Milde S., Vilalta A., Cockram T.O.J., Allendorf D.H., Lee J.Y., Dundee J.M., Pampuščenko K., Borutaite V., Nuthall H.N. (2021). The microglial P2Y6 receptor mediates neuronal loss and memory deficits in neurodegeneration. Cell Rep..

[62] Dejanovic B., Huntley M.A., De Mazière A., Meilandt W.J., Wu T., Srinivasan K., Jiang Z., Gandham V., Friedman B.A., Ngu H. (2018). Changes in the Synaptic Proteome in Tauopathy and Rescue of Tau-Induced Synapse Loss by C1q Antibodies. Neuron.

[63] Jürgens T., Jafari M., Kreutzfeldt M., Bahn E., Brück W., Kerschensteiner M., Merkler D. (2016). Reconstruction of single cortical projection neurons reveals primary spine loss in multiple sclerosis. Brain.

[64] Schwarz K., Schmitz F. (2023). Synapse Dysfunctions in Multiple Sclerosis. Int. J. Mol. Sci..

[65] Carassiti D., Altmann D.R., Petrova N., Pakkenberg B., Scaravilli F., Schmierer K. (2018). Neuronal loss, demyelination and volume change in the multiple sclerosis neocortex. Neuropathol. Appl. Neurobiol..

[66] Werneburg S., Jung J., Kunjamma R., Ha S., Luciano N., Willis C., Gao G., Biscola N.P., Havton L.A., Crocker S.J. (2020). Targeted complement inhibition at synapses prevents microglial synaptic engulfment and synapse loss in demyelinating disease. Immunity.

[67] Ramaglia V., Dubey M., Malpede M.A., Petersen N., de Vries S.I., Ahmed S.M., Lee D.S.W., Schenk G.J., Gold S.M., Huitinga I. (2021). Complement-associated loss of CA2 inhibitory synapses in the demyelinated hippocampus impairs memory. Acta Neuropathol..

